# Usefulness of deep sedation with intravenous dexmedetomidine and midazolam in cardiac catheterization procedures for pediatric patients

**DOI:** 10.3389/fped.2024.1338130

**Published:** 2024-08-15

**Authors:** Taichi Nakamura, Hidenori Iwasaki, Hanae Miyazawa, Shinichiro Mizutomi, Yoko Imi, Kunio Ohta, Taizo Wada

**Affiliations:** ^1^ Department of Pediatrics, School of Medicine, Institute of Medical, Pharmaceutical and Health Sciences, Kanazawa University, Kanazawa, Japan; ^2^ Medical Education Research Center, Graduate School of Medical Sciences, Kanazawa University, Kanazawa, Japan

**Keywords:** cardiac catheterization, dexmedetomidine, hypoxemia, midazolam, sedation

## Abstract

**Background:**

Dexmedetomidine (DEX) is a highly selective alpha 2 receptor agonist that has the advantage of causing less respiratory depression than other sedative agents. We evaluated the add-on effects of DEX on sedation among pediatric patients who received midazolam and pentazocine during cardiac catheterization.

**Methods:**

120 cardiac catheterization procedures in 110 patients under deep sedation at Department of Pediatrics, Kanazawa University Hospital from January 2013 to August 2018: 63 procedures without DEX (i.e., non-DEX group) and 57 procedures with DEX (i.e., DEX group). Intravenous midazolam and pentazocine were used in both groups, and DEX without an initial loading dose (0.6 μg/kg/h) was used in the DEX group. We retrospectively investigated complications during catheterization, doses of sedative agents, and changes in vital signs.

**Results:**

Hypoxemia requiring oxygen administration during catheterization tended to be higher in the non-DEX group than in the DEX group (4.8% vs. 0%). Additional dose of midazolam was significantly lower in the DEX group (median [IQR]: 0.05 mg/kg [0–0.11]) than in the non-DEX group [0.09 mg/kg (0–0.23), *p* = 0.0288]. The additional dose of midazolam in the non-DEX group with hypoxemia was significantly higher than the dose used in the non-DEX group without hypoxemia. No case of bradycardia below the criteria for bradycardia occurred and no serious complications occurred in the DEX group.

**Conclusion:**

The use of intravenous DEX in combination with midazolam and pentazocine in pediatric cardiac catheterization may reduce the need for an additional dose of midazolam and may contribute to the prevention of airway complications associated with respiratory depression caused by sedative agents.

## 1 Introduction

Control of patient's body movements is a critical aspect of accurate and safe examination during pediatric cardiac catheterization in which sedative agents are commonly used during painful invasive procedures (1). Hemodynamic data from patients administered general anesthesia and positive pressure ventilation are less accurate than data from nonintubated spontaneously breathing patients. Therefore, medical professionals who are involved in pediatric anesthesia need to select appropriate anesthetics which can achieve accurate results without serious adverse events during nonintubated catheterization.

Midazolam is a classic sedative agent administered to patients undergoing invasive procedures (2). Intravenous midazolam has minimal hemodynamic effects; however, it can cause loss of airway reflexes, respiratory depression, and apnea. Dexmedetomidine (DEX) is a potent and highly selective alpha 2 receptor agonist with sedative, analgesic, and anxiolytic effects (3). Its adverse effects include bradycardia and hypotension; however, intravenous DEX has the advantage of causing less respiratory depression than other sedative agents, including midazolam. DEX is useful as a sedative for noninvasive procedures such as magnetic resonance imaging scans in pediatric patients (4). However, intravenous DEX alone is insufficient to provide adequate sedation for patients undergoing invasive procedures such as cardiac catheterization (5); thus, it is combined with other sedative agents. No previous reports have examined the usefulness of the combination of intravenous midazolam and DEX in sedation for pediatric cardiac catheterization. The purpose of this study was to investigate the add-on effects of standard-dose DEX without an initial loading dose among patients under sedation with midazolam and pentazocine, a potent analgesic, for cardiac catheterization.

## 2 Materials and methods

### 2.1 Study design

Of the 185 cardiac catheterization procedures for pediatric patients at our hospital from January 2013 to August 2018, 120 procedures in 110 patients, including six adult patients (age range, 18–28 years), under deep sedation, as described below, were included. Procedures with general anesthesia, with thiopental administration, or without sufficient data were excluded. Cardiac catheterizations with simultaneous transesophageal echocardiography were also excluded because this procedure may affect hemodynamic parameters. The procedures were divided into two groups, based on whether they were performed under sedation with DEX or without DEX, which was routinely used after July 2015: 63 procedures in 60 patients without DEX use (i.e., non-DEX group) and 57 procedures in 54 patients with DEX use (i.e., DEX group). We retrospectively evaluated complications during catheterization, the dose of sedative agents, and changes in vital signs in both groups. All patients received 1 mg/kg of intravenous hydroxyzine hydrochloride as a premedication 30 min before going to the catheterization room. Sedative agents that were administered were midazolam (dose range, 0.1–0.2 mg/kg) and pentazocine (dose range, 0.3–0.5 mg/kg) with monitoring after entering the catheterization room. DEX (dose, 0.6 μg/kg/h) was administered without an initial loading dose just before the start of the procedure in the DEX group. The infusion rate of DEX was reduced by half so that the heart rate did not decrease below the criteria for bradycardia based on the decision by pediatric cardiologists. We did not increase the infusion rate of DEX during catheterization. Local anesthesia with 0.5% lidocaine was administered at the time of sheath insertion. Midazolam was added at a dose of 0.05–0.1 mg/kg each time when there was difficulty in controlling patient's body movements (maximum total dose: 1.0 mg/kg). Pentazocine was used only for induction of anesthesia and additional dose of pentazocine was not administered unless painful procedures were performed (maximum total dose: 0.5 mg/kg). Patients were managed under spontaneous breathing during catheterization by one pediatric cardiologist. Hypoxemia was defined as a persistent decline of percutaneous oxygen saturation (SpO_2_) below 90% which did not improve with airway management using mandibular elevation. In the condition presenting with right-to-left shunt like tetralogy of Fallot, hypoxemia was defined as a decrease in SpO2 greater than 10% compared with the SpO_2_ just before the induction of anesthesia (i.e., basal SpO2) which did not improve with the same airway management. Oxygen administration was performed when the pediatric cardiologist decided that hypoxemia which met the criteria occurred. Informed consent for sedation with DEX was obtained from patients or parents before catheterization, and the study was approved by our institutional review board.

### 2.2 Data collection and analysis

Patient background was investigated in terms of age, sex, height, weight, body surface area, diagnosis, basal SpO_2,_ and previous surgery. In addition, data were collected on cardiac catheterization time, whether a catheter intervention was or was not performed, complications during catheterization, and sedative use. Doses per body weight were calculated for midazolam and pentazocine. For midazolam, data were collected on the initial dose, additional dose, and total dose; the DEX dose was examined in the DEX group.

Vital signs were measured non-invasively every 5 min after the induction of sedation. Basal SpO2 was defined as the SpO2 just before the induction of anesthesia. The changes in heart rate (HR), systolic blood pressure (BPs), and mean blood pressure (BPm) during procedures were compared between the DEX and non-DEX groups. The changes in HR were calculated as the differences between the lowest HR during catheterization and the HR just before the induction of anesthesia. The baseline of BPs and BPm is defined as BP immediately after the start of sedation since there was not enough data about BP available after premedication or before the induction of sedation. Based on the criteria used in previous reports (6–8), the definition of bradycardia by age was set at <100 bpm for neonates, <80 bpm for one month to <2 years, <60 bpm for 2–6 years, <45 bpm for 6–11 years, and <40 bpm for >11 years.

### 2.3 Statistical analysis

Statistical analysis was conducted using GraphPad Prism ver. 8.00 for Mac (GraphPad Software, San Diego, CA, USA). Statistical analysis of differences between the two groups with regard to age, height, weight, body surface area, cardiac catheterization time, basal SpO_2_, HR, BPs, BPm, and the dose of sedatives was conducted using the Mann-Whitney *U* test. Data regarding the presence of intervention, sex, diagnosis, surgical history, and complications during catheterization were analyzed with the v2 test (i.e., Fisher's exact test). Differences between groups with a *p* value <0.05 were statistically significant.

## 3 Results

### 3.1 Patient background and clinical characteristics

The clinical characteristics are shown in Table 1. No statistically significant differences existed in age, sex, weight, height, basal SpO_2_ or surgical history between the DEX and non-DEX groups. No differences also existed between the two groups in the time required for catheterization or the number of interventions performed. The two groups had similar proportions of congenital heart disease and coronary artery aneurysms after Kawasaki disease.

**Table 1:** Clinical data of the dexmedetomidine and non-dexmedetomidine groups.

	DEX group (*N* = 57)	Non-DEX group (*N* = 63)	*p* value
Age at catheterization (months)	39 [7–73]	45 [11–71]	0.443
Sex	38M (67%) /19F (33%)	37M (59%) /26F (41%)	0.451
Surgical history	13 (23%)	16 (25%)	0.832
Weight (kg)	13.1 [6–19.1]	13.1 [7.8–21.7]	0.361
Height (cm)	89.8 [64–113]	93.5 [70.8–116]	0.444
Body surface area (m^2^)	0.56 [0.32–0.77]	0.58 [0.39–0.8]	0.350
Catheterization time (min)	90 [72–115]	90 [75–106]	0.638
Intervention	4 (7%)	3 (5%)	0.707
SpO_2_ just before the induction of anesthesia (%)	96 [95–98]	97 [95–98]	0.184
Diagnosis			
VSD	16 (28%)	21 (33%)	0.559
Post-KD	12 (21%)	8 (13%)	0.233
ASD	2 (4%)	2 (3%)	>0.999
PDA	6 (11%)	7 (11%)	>0.999
TOF	3 (5%)	6 (10%)	0.496
Other	18 (32%)	19 (30%)	>0.999

The data are presented as median [IQR] or as the number (percent).

DEX, dexmedetomidine; M, Male; F, Female; SpO_2_, percutaneous oxygen saturation; VSD, ventricular septal defect; KD, Kawasaki disease; ASD, atrial septal defect; PDA, patent ductus arteriosus; TOF, tetralogy of Fallot.

### 3.2 Complications during catheterization

As shown in Table 2, hypoxemia requiring oxygen administration during catheterization tended to be higher in the non-DEX group than in the DEX group (4.8% vs. 0%), but this difference was not significant. Details of the characteristics of each procedure with hypoxemia in three patients in the non-DEX group are described in Table 3. Supraventricular tachycardia was absent from all patients in the DEX group but was present only in one (1.6%) patient in the non-DEX group. We observed bradycardia in one (1.6%) patient in the non-DEX group, who had an underlying sick sinus syndrome (Table 2).

**Table 2:** Complications occurring during catheterization.

	DEX group (*N* = 57)	Non-DEX group (*N* = 63)	*p* value
Hypoxemia requiring oxygen	0 (0%)	3 (4.8%)	0.246
SVT	0 (0%)	1 (1.6%)	>0.999
Bradycardia	0 (0%)	1 (1.6%)	>0.999

The data are presented as the number (percent).

DEX, dexmedetomidine; SVT, supraventricular tachycardia.

**Table 3:** Characteristics of the three patients with hypoxemia requiring oxygen.

Age at catheterization (months)	Diagnosis	MDZ dose (mg/kg)			DEX dose (μg/kg/h)
		Initial	Additional	Total	
9	TOF	0.13	0.42	0.55	–
9	TOF	0.14	0.29	0.43	–
63	VSD	0.14	0.23	0.36	–

MDZ, midazolam; TOF, tetralogy of Fallot; VSD, ventricular septal defect.

### 3.3 Sedative dosage

**Figure 1:**
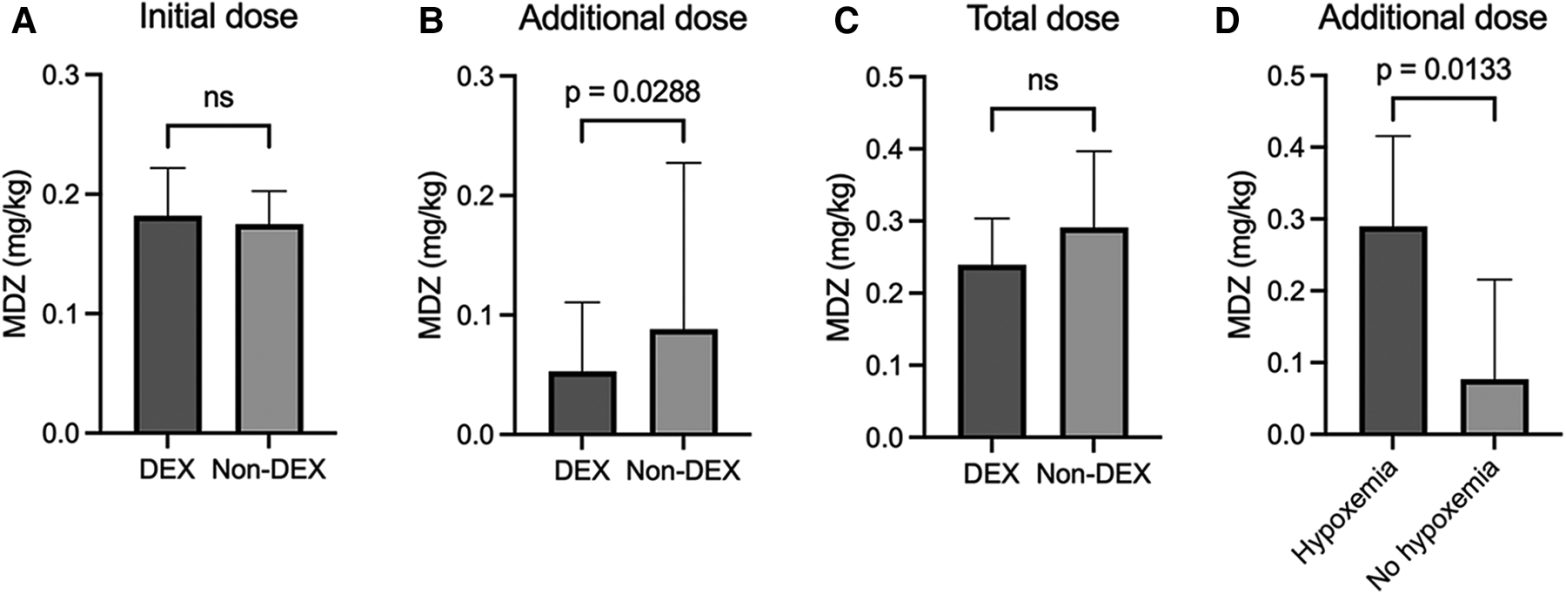
Initial **(A)**, additional **(B)**, and total dosage **(C)** of midazolam in the dexmedetomidine and non-dexmedetomidine groups. **(D)** Comparison of additional midazolam doses with and without hypoxemia in the non-dexmedetomidine group. The values are presented as median [IQR]. MDZ, midazolam; DEX, dexmedetomidine; ns, not significant.

The initial dose of midazolam was not different between the DEX group [0.18 mg/kg (0.13–0.22)] and non-DEX group [0.18 mg/kg (0.11–0.20)] (Figure 1A). The dose of pentazocine also did not differ between the two groups (DEX group: 0.40 mg/kg [0.34–0.46]; non-DEX group: 0.41 mg/kg [0.33–0.49]). DEX was used only in the DEX group [initial DEX dose, 0.59 μg/kg/h (0.55–0.60)]. However, the additional dose of midazolam was significantly lower in the DEX group [0.05 mg/kg (0–0.11)] than in the non-DEX group [0.09 mg/kg (0–0.23), *p* = 0.0288] (Figure 1B). The total dose of midazolam tended to be lower in the DEX group (DEX group: 0.24 mg/kg [0.18–0.30]; non-DEX group: 0.29 mg/kg [0.16–0.40]), but the difference was not significant (Figure 1C).

To elucidate further the characteristics of the additional usage of midazolam in the non-DEX group, these procedures were divided into two subgroups, based on the presence of hypoxemia requiring oxygen administration. Figure 1D clearly demonstrates that the additional dose of midazolam in the non-DEX group with hypoxemia was significantly higher than the dose used in the non-DEX group without hypoxemia (0.29 mg/kg [0.23–0.42] vs. 0.08 mg/kg [0–0.22], *p* = 0.0133).

### 3.4 Changes in vital signs during catheterization

**Figure 2:**
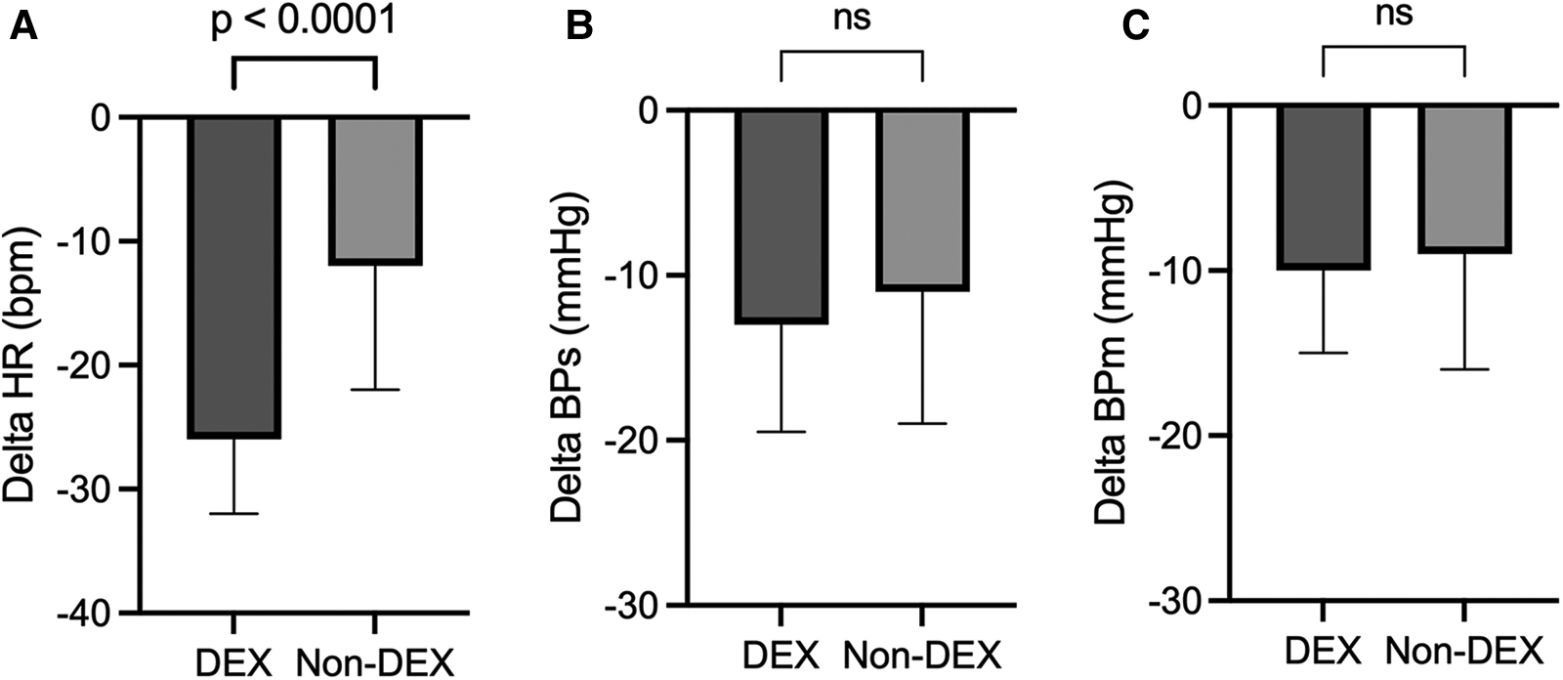
Changes in heart rate **(A)**, systolic blood pressure **(B)**, and mean blood pressure **(C)** during catheterization. The values are presented as median [IQR]. DEX, dexmedetomidine; ns, not significant; HR, heart rate; BPs, systolic blood pressure; BPm, mean blood pressure.

Changes in HR, BPs, and BPm during catheterization were shown in Figure 2. The delta HR (i.e., the lowest HR during catheterization, compared with the HR just before the induction of anesthesia) was significantly larger in the DEX group {−26 bpm [−32 to (−20)]} than non-DEX group {−12 bpm [−22 to (−7)], *p* < 0.0001} (Figure 2A). The delta BPs during catheterization (DEX group: −13 mmHg [−20 to (−9)]; non-DEX group: −11 mmHg [−19 to (−5)]) and the delta BPm (DEX group: −10 mmHg [−15 to (−6)]; non-DEX group: −9 mmHg [−16 to (−5)]) tended to be larger in the DEX group, but no significant differences existed between the two groups (Figures 2B, C).

## 4 Discussion

This study demonstrated that intravenous midazolam plus pentazocine used in combination with DEX can provide adequate sedation and significantly reduce the additional doses of midazolam during pediatric cardiac catheterization. Midazolam is prone to inducing hypoxemia due to respiratory depression. Hypoxemia increases the burden on the patient and affects hemodynamic parameters. Therefore, minimizing the dosage of midazolam is desirable. However, accurate data cannot be obtained without control of patient's body movements and stable breathing; therefore, deep sedation with higher dose of midazolam may be required during catheterization. A possible solution to this dilemma is the combined use of midazolam with DEX, which is reported to cause less respiratory depression (9). Achieving strong sedation for invasive procedures is difficult when using DEX as a single agent (10); however, DEX can provide stable sedation when used in combination with other sedatives (11). In fact, the combined use of DEX has also been reported to decrease the use of other anesthetics, including propofol, thereby resulting in fewer complications associated with respiratory depression (12, 13). These findings are consistent with our results demonstrating that the combination of DEX reduced the additional midazolam dose in the non-DEX group by an average of 0.06 mg/kg, which is equivalent to approximately 46% of the additional midazolam dose in the non-DEX group. The additional dose of midazolam in the non-DEX group with hypoxemia was significantly higher than the dose used in the non-DEX group without hypoxemia; therefore, a reduction in sedative agents is ideal for reducing complications. Sedation with midazolam alone has been reported to cause hypoxemia in 5.8% of nonintubated pediatric patients undergoing invasive procedures (14), which is similar to the rate of hypoxemia in the non-DEX group in this study. Although the frequency of hypoxemia was not significantly different between the DEX and non-DEX groups, sedation with the combined use of DEX tended to cause less hypoxemia requiring oxygen administration.

In Europe and in the United States, propofol is often combined with ketamine or fentanyl for the sedation of children undergoing highly invasive procedures. However, in Japan, midazolam is often used for sedation outside of the operating room because of the concern that propofol has adverse effects such as propofol infusion syndrome and strong vasodilation. Midazolam may be used alone or in combination with other sedative agents. In fact, midazolam plus DEX has reportedly been used in the postoperative management of cardiac surgery in children (15). However, the effectiveness of the combination of midazolam and DEX in cardiac catheterization has not been reported.

By contrast, sedation with DEX may be performed with DEX alone or in combination with other agents. If sedation is performed with DEX alone, a maintenance dose is usually given after the initial loading (16). DEX has been used in combination with other sedative agents under the condition of loading (12, 17) and without loading (18). We chose low-dose DEX without a loading condition (18) and achieved adequate sedation for cardiac catheterization.

DEX has the adverse effects of decreasing HR and blood pressure. In fact, hypotension with DEX administration has been reported in healthy adult volunteers (19). Therefore, low-dose DEX can be expected to reduce the frequency of hypotension. In fact, no significant difference existed in the change in BPs and BPm between the DEX and non-DEX groups in this study. Despite receiving a low dose of DEX, procedures with DEX administration presented with a significant reduction in HR during catheterization. However, bradycardia which meet the criteria did not occur in the DEX group when the dose of DEX was properly adjusted. Furthermore, other adverse events which were suspected to be related with the use of DEX were also not observed. Monitoring the patients' vital signs during sedation is extremely important regardless of the concomitant use of DEX; however, attention should be paid particularly to bradycardia during the sedation with DEX, even when the low dose of DEX was used. Taken together, the combination of midazolam, pentazocine, and DEX may be as safe as midazolam plus pentazocine for sedation during pediatric cardiac catheterization.

This study has limitations. Selection bias exists because of the fact that this study was a retrospective study of a single racial group at a single institution. Additionally, variability in HR was observed because of the wide age range, although HR just before the induction of sedation did not differ between the DEX group [125 bpm (103–143)] and non-DEX group [120 bpm (94–136), *p* = 0.241], which may have had little influence on the results of this study. Furthermore, a previous report described that the monitoring of end-tidal CO_2_, i.e., an alternative indicator of PaCO_2_, could detect subclinical respiratory depression (20). However, we could not evaluate CO_2_ retention as a critical indicator of respiratory depression because we did not routinely measure PaCO_2_ unless rigorous monitoring of respiratory status was required.

## 5 Conclusions

The use of intravenous DEX in combination with midazolam and pentazocine may reduce the need for an additional dose of midazolam in deep sedation during pediatric cardiac catheterization and may contribute to the prevention of airway complications associated with respiratory depression during sedation.

## Data Availability

The original contributions presented in the study are included in the article/Supplementary Material, further inquiries can be directed to the corresponding author.
